# In Vivo Performance of Decellularized Vascular Grafts: A Review Article

**DOI:** 10.3390/ijms19072101

**Published:** 2018-07-19

**Authors:** Chih-Hsun Lin, Kai Hsia, Hsu Ma, Hsinyu Lee, Jen-Her Lu

**Affiliations:** 1 Division of Plastic Surgery, Department of Surgery, Taipei Veterans General Hospital, Taipei 11217, Taiwan; chlin12@vghtpe.gov.tw (C.-H.L.); sma@vghtpe.gov.tw (H.M.); 2 Department of Surgery, School of Medicine, National Yang-Ming University, Taipei 11221, Taiwan; 3 Department of Pediatrics, Taipei Veterans General Hospital, Taipei 11217, Taiwan; hkay1008@gmail.com; 4 Department of Life Science, National Taiwan University, Taipei 10617, Taiwan; hsinyu@ntu.edu.tw; 5 Department of Surgery, School of Medicine, National Defense Medical Center, Taipei 11490, Taiwan; 6 Department of Pediatrics, School of Medicine, National Yang-Ming University, Taipei 11221, Taiwan; 7 Department of Medicine, School of Medicine, National Defense Medical Center, Taipei 11490, Taiwan; 8 Department of Pediatrics, School of Medicine, National Defense Medical Center, Taipei 11490, Taiwan

**Keywords:** vascular graft, decellularization, recellularization, animal model

## Abstract

Due to poor vessel quality in patients with cardiovascular diseases, there has been an increased demand for small-diameter tissue-engineered blood vessels that can be used as replacement grafts in bypass surgery. Decellularization techniques to minimize cellular inflammation have been applied in tissue engineering research for the development of small-diameter vascular grafts. The biocompatibility of allogenic or xenogenic decellularized matrices has been evaluated in vitro and in vivo. Both short-term and long-term preclinical studies are crucial for evaluation of the in vivo performance of decellularized vascular grafts. This review offers insight into the various preclinical studies that have been performed using decellularized vascular grafts. Different strategies, such as surface-modified, recellularized, or hybrid vascular grafts, used to improve neoendothelialization and vascular wall remodeling, are also highlighted. This review provides information on the current status and the future development of decellularized vascular grafts.

## 1. Introduction

### 1.1. Rationale for the Use of Decellularized Vascular Grafts

Decellularized biological scaffolds from vascular tissue derived either from allogenic or xenogenic sources have the potential to serve as substitutes for artificial vascular conduits, which would help solve the issues of donor vessel shortage and immune reaction in the recipient. Decellularized vascular grafts that are currently on the market include products such as Artegraft^®^ (Bovine carotid artery), Solcograft (Bovine carotid artery), ProCol^®^ (Bovine mesenteric vein), and SynerGraft^®^ (Bovine ureter); these products are available for clinical use [1,2]. Most of these grafts are used for vascular access during hemodialysis or peripheral arterial bypass when a relatively large diameter is needed. However, clinical outcomes are not satisfactory due to graft-related thrombosis, infection, and aneurysm. At present, decellularized xenogenic grafts offer only non-inferior results compared with alternative synthetic conduits. For example, early results of studies using Synergraft^®^ have revealed the risk for aneurysm formation, poor long-term patency, infection, and inflammation [3,4,5]. The limited performance of commercially available decellularized vascular grafts has been suggested to be due to their lack of cellularity upon implantation [6]. Another disadvantage of current decellularized xenogeneic grafts is their higher cost compared with synthetic grafts. Thus, they have not been widely used in the clinic [2]. Hence, various decellularized matrices derived from the human umbilical artery, human umbilical vein, porcine carotid artery, porcine radial artery, porcine saphenous artery, porcine iliac artery, small intestine submucosa (SIS), and bovine ureter [7,8,9,10,11,12,13,14] have been continuously investigated for application in small-diameter vascular tissue engineering.

Decellularized biological scaffolds are composed of extracellular matrix (ECM), primarily collagen, elastin, and glycosaminoglycan (GAG), and can provide a surface that is abundant in the cell signaling components that are essential for cell adhesion, migration, proliferation, and differentiation [15]. For vascular tissue, the collagen and elastin fibers in the medial layer provide elasticity to withstand pulsatile blood flow within a physiological range. Of these ECM components, collagen is responsible for the retention of tensile strength, elastin fibers maintain the elastic properties of the scaffold, and GAGs provide viscoelasticity [16]. The predominant cells in the tunica intima and tunica media of vascular tissue are endothelial cells (ECs) and smooth muscle cells (SMCs), respectively. The endothelium not only prevents thrombosis but is also responsible for the maintenance and alterations in the vascular tone, which controls blood flow. SMCs are present in the medial layers of the vessels, circumferentially, along with collagen and elastin fibers, which provide mechanical support. SMCs possess contractile properties that are required for expansion and contraction as blood flows; they also produce the ECM to replenish the degraded fibers. Fibroblasts are located primarily in the adventitia, along with large bundles of collagen fibers that function in longitudinal mechanical support. After the removal of cells, the decellularized scaffolds that constitute the ECM can maintain the mechanical properties of natural vessels to withstand changes in blood pressure. After implantation, the natural matrix induces tissue remodeling and regeneration.

Decellularization is typically accomplished by treating tissues with a combination of freeze–thawing, osmotic gradients, solvents, detergents, enzymes, chelating agents, and physical methods [17]. For example, high hydrostatic pressure (HHP) is used for the decellularization of vascular tissue [10,18]. The efficiency of decellularization is usually initially evaluated by histological examinations such as H&E staining to determine the residual number of cells in the tissues [19]. Special stains such as Verhoeff-Van Gieson, Masson’s trichrome, Movat’s Pentachrome, or Safrin O can be used to examine tissues for the presence of various cytoplasmic and extracellular molecules [19]. Immunohistochemical methods are utilized to detect specific intracellular proteins, such as α-actin and vimentin as well as adhesion-related proteins, such as fibronectin and laminin [20]. The presence of DNA can be evaluated by DAPI or Hoechst staining. Propidium iodide and PicoGreen have been developed to provide a quantitative DNA assessment [21]. Polymerase chain reaction (PCR), Western blot, and electron microscopy are adjunctive methods that may be used to determine the presence of remnant nuclear material or cytoplasmic debris. Biochemical assays are available to confirm that desirable components of the ECM, such as elastin, collagen, and GAGs, are still present [17]. Mechanical testing of the ECM after treatment provides insight into the presence and integrity of the structural proteins within the scaffold [19,22].

Notably, the concentration and structure of ECM proteins in the scaffold will change after decellularization. This alteration in ECM structure, which depends greatly on the decellularization process, will also lead to changes in the mechanical properties [23]. For example, prolonged exposure of the tissue to trypsin during decellularization may disrupt the ECM structure, remove biomolecules, and change the mechanical properties [24,25]. Studies on the use of scattered-angle light spectroscopy and multi-photon microscopy have indeed shown a change in fiber alignment, distribution, and motility of ECM proteins after decellularization [23,25]. In our mathematic model analysis of decellularized porcine coronary artery, we also noticed a gradual transition from anisotropic to isotropic in the uniaxial tensile properties of porcine coronary artery during trypsin-based decellularization. This alteration was correlated with a degradation of elastin and a decrease in GAGs in the ECM [26]. Hence, optimal decellularization is crucial for vascular grafts because inappropriate decellularization of vascular grafts may result in early intimal hyperplasia, graft occlusion, failure, or aneurysmal dilatation after implantation in vivo [27].

### 1.2. In Vitro Studies of Decellularized Vascular Grafts

In vitro, it has been shown that decellularized biological scaffolds can support endothelial cell adhesion and growth. For example, Dahl et al. demonstrated the feasibility of re-seeding decellularized vessels with vascular cells [28]. Gui et al. found that decellularized umbilical arteries supported endothelial adherence as indicated via re-endothelialization by a monolayer of human umbilical vein endothelial cells (HUVECs) [7]. Xiong et al. revealed the in vitro cell adhesion and biocompatibility of decellularized porcine saphenous artery and showed that porcine pulmonary artery endothelial cells were able to adhere and proliferate on scaffolds in both static and rotational culture [11]. Decellularized biological scaffolds can also support smooth muscle cell adhesion and growth. Fitriatul et al. used 2% sodium dodecyl sulfate (SDS) and sonication decellularization treatment for an aortic scaffold and demonstrated successful SMC repopulation [29]. Furthermore, decellularized biological scaffolds maintain endothelial and smooth muscle cell differentiation status and phenotype. Schneider et al. demonstrated that HUVECs cultured on decellularized placenta vessel matrix underwent endothelialization and maintained appropriate phenotypic characteristics and cell-specific expression patterns [30]. Dahan et al. showed that a decellularized arterial matrix supported repopulation with endothelial and smooth muscle cells after dynamic culture. In addition, smooth muscle cells expressed high initial levels of remodeling-related genes, which were concomitantly expressed along with an increased secretion of GAGs [31]. By supporting endothelial cell growth, decellularized biological scaffolds can reduce thrombogenicity and restore vascular function. Uzarski et al. found that, in vitro, decellularized human umbilical vein scaffolds were capable of being repopulated by viable endothelial cells, while they were also resistant to excessive early adhesion by platelets and inflammatory cells [32]. Barder et al. re-seeded decellularized porcine aorta with human endothelial cells and myofibroblasts in a bioreactor. The 3D matrix was covered with a fully confluent layer of human endothelial cells, and myofibroblasts migrated into positions formerly occupied by xenogeneic cells. In addition, maintenance of nitric oxide synthase activity was noted in the bioartificial graft [33]. The positive effects of decellularized vascular grafts on endothelial cell retention, adhesion, proliferation and differentiation might be related to biomolecules or growth factors inside the grafts. For example, the small intestinal submucosa, which retains angiogenic growth factors, such as basic fibroblast growth factor (bFGF) and vascular endothelial growth factor (VEGF), has been shown to support human endothelial cell retention, adhesion and proliferation. Recently, Khan et al. revealed that FGF and VEGF promote adipose stem cell (ASC) proliferation, migration, attachment, and endothelial differentiation in SIS [33,34]. Conklin et al. also showed that heparin-linked decellularized porcine carotid arteries can reduce thrombogenicity in vitro [8].

Of particular importance for vascular grafts are binding interactions between the biomaterial and host cells from the circulation and adjacent vasculature once implantation occurs in vivo [35]. As decellularized donor tissues have shown great promise for cell adhesion in vitro, they are expected to finally become a functionalized vascular conduit once they are implanted. Early experimental results with a 52-week graft patency were observed in vivo when a decellularized biological scaffold (SIS) was implanted as a large diameter vena cava vascular graft in canines [36]. Continuing studies have focused on the application and performance of decellularized vascular grafts in vivo over both the short term and the long term. Despite the existence of cell-free decellularized vascular grafts without further treatment, in order to improve neoendothelialization, surface modification or recellularization techniques have also been investigated and have been recently applied to decellularized vascular grafts. With more results from animal studies, we expect to learn the future of this type of biomaterial.

### 1.3. Biomolecules and Growth Factors

To improve the patency of implanted vascular grafts, early confluent endothelialization at the luminal surface of the graft and avoidance of thrombus formation are critical steps. Heparin is well known to prevent thrombosis due to its inhibition of the thrombin clotting cascade, and therefore, it has been applied in vascular tissue engineering as a heparin coating or binding that activates anti-thrombin II (ATII) [37,38]. Heparin can bind to polymers via ionic, electrostatic or covalent interactions. However, Koobatian et al. reported that immobilized heparin was insufficient for the maintenance of long-term patency and required the presence of VEGF, which was bound via the heparin binding domain [13]. In addition to preventing thrombus formation, reconstitution of the endothelium should occur as quickly as possible once implantation is completed and blood flow has been established. The endothelium in implanted decellularized vascular grafts may be derived from native endothelial cells that migrate from peri-anastomotic sites, transmural ingrowth of capillaries or from the recruitment of circulatory endothelial progenitor cells (EPCs) [39,40]. Heparin-coated, fast-degrading elastomers have also been evaluated in rats and have been shown to be effective in endothelialization as decellularized grafts [41]. In addition, immobilization of VEGF on surface-bound heparin enabled the highly selective capture of endothelial cells (ECs) under low (0.5 dyne/cm^2^) and high (15 dyne/cm^2^) shear stress, even from complex biological fluids such as blood [42]. Previous studies also demonstrated that treatment with biomolecules/growth factors, such as granulocyte-colony stimulating factor (G-CSF), brain derived growth factor (BDGF), cysteine-rich protein (CYR61; CCN1) and stromal cell derived factor (SDF-1α), was beneficial for graft patency and perhaps also for remodeling [43,44,45,46]. For example, SDF-1α enabled recruitment of EPCs to the luminal surface of the graft, with improved the long-term patency of synthetic vascular grafts in vivo [45]. Our group also demonstrated that sphingosine-1-phosphate (S1P) enhances attachment of HUVECs and EPCs. S1P-treated HUVECs that recellularized vascular grafts showed inhibition of platelet adherence and reduced thrombus formation by protecting Syndecan-1 (SDC1) from shedding [47]. Recently, antihypertensive drugs such as losartan have been demonstrated to improve medial layer regeneration and to decrease elastin degradation of decellularized vascular grafts in a rat model [48]. Nanotechnology has also been applied to enhance endothelialization of decellularized vascular grafts. Tan et al. used chitosan/heparin nanoparticles to control the release of VEGF on decellularized vascular grafts. This approach improved endothelial cell proliferation in vitro [49]. Shimizu et al. successfully recellularized porcine decellularized vascular grafts with human cells using magnetite nanoparticle technology [50].

### 1.4. Recellularization Techniques

It has been shown that cell seeding may play a role in the maintenance of vascular graft patency [9,51]. Endothelialization of the vasculature is necessary to prevent thrombosis during transplantation as any exposed collagen will activate platelets and the extrinsic coagulation cascade, which may contribute to unsuccessful transplantation. However, mixed results were obtained regarding biodegradable polymer scaffolds. Some studies have shown that cell (ECs and SMCs)-seeded vascular grafts developed thrombosis shortly after implantation [52,53]. Mirensky et al. found that bone marrow-monocyte-seeded synthetic vascular grafts contained a luminal endothelial lining surrounded by a concentric smooth muscle cell layer and collagen after a 6-month implantation in murine inferior vena cava [54].

Cell-free decellularized vascular grafts without further treatment have been shown to be effective in large animal models and even in clinical settings, but host cell infiltration was limited near the anastomotic sites, and as a result, remodeling was also limited to those sites [28]. For decellularized vascular grafts, a functional endothelium may still be required to maintain patency [20]. Increasing numbers of researchers have begun to use EPCs or multipotent stem cells for vascular graft recellularization. The disadvantage of recellularization is the potentially high associated cost due to several weeks or months of preparation, such as the culture of autologous smooth muscle cells (SMCs) and ECs, which are required for these grafts to be ready for implantation. The key parameters in the recellularization process include cell type, cell number, seeding methods and culture environment. Vascular grafts may be seeded with relevant cell types such as ECs, SMCs or EPCs and may be allowed to mature before implantation [55,56]. At present, no consensus has been reached as to the appropriate cell number or cell density (10^5^~10^7^ cells/mL) for re-seeding. Different seeding/culture methods to repopulate the decellularized vascular scaffold in order to facilitate robust endothelialization have also been explored. Two main types of recellularization techniques are the static and dynamic methods [51,57]. The static method entails the direct dripping/injection/bathing of cells into the luminal or abluminal side of the graft and then cultivation under static conditions [27,51,58]. This method is simple and less expensive but is usually criticized because of low efficiency. Bergmeister et al. used a laser perforation technique to allow early recellularization of decellularized allogenic vascular grafts in sheep carotid artery transplantation model [59]. Recently, Książek et al. proposed a “puncturing-dripping” method for re-seeding tissue-engineered vascular matrices. The results demonstrated that the highest cell-loading efficiency was observed after lyophilization followed by the puncturing-dripping technique [60]. A dynamic culture system typically involves a pulsatile perfusion bioreactor to simulate blood flow/flow rate in vivo [61]. Dynamic seeding techniques increase cell seeding efficiency, improve uniformity and enhance cell penetration of the scaffold [51]. Biomechanical stimuli serve as integral components in the development of a mature endothelium. Mechanical forces such as shear stress, which are applied by blood flow, can affect vascular remodeling, homeostasis, and disease [62]. Two main kinds of dynamic culture are rotational seeding and vacuum seeding. Rotational systems induce hydrostatic forces and vacuum systems create pressure gradients [63,64,65,66]. Dynamic culture can offer distinct advantages over static culture conditions, including cell waste removal, nutrient delivery, and mechanical stimuli [67]. More recent work has also demonstrated the importance of cell-to-cell interactions in the recellularization process [31,68]. However, another study showed that the presence of pre-seeded SMCs in the vascular wall of SIS-fibrin hybrid grafts enhanced, but was not necessary for, host cell infiltration, remodeling and development of vascular function [69].

Alternative sources to differentiated vascular cells or smooth muscle cells, including stem cells, have been explored. Stem cells are capable of self-renewal and differentiation into mature cells under proper conditions. Some advantages of adult stem cells are the possibility of obtaining large numbers of functional cells, avoidance of immune rejection and ethical problems, such as avoiding destruction of early human embryos, and lower tumorigenic capacity [70]. Mesenchymal stem cells (MSCs), pericytes, circulatory or adipose tissue progenitor cells and induced pluripotent stem (iPS) cells show great potential for use in tissue engineering applications and may circumvent many of the shortcomings associated with other methods of cell sourcing [71,72]. For example, MSCs are a promising cell type for regenerative medicine due to their ease of isolation and expansion, multipotency and low immunogenicity [73]. Pericytes have recently been found to exhibit mesenchymal stem cell features. The relative availability and multipotentiality make this cell type a promising candidate for TEVG applications [74]. EPCs are easy to obtain from a patient who requires the procedure and are easy to expand in culture. Indeed, EPCs can be expanded for over 20 passages without loss of their differentiation potential [75]. These cells are primarily located in the bone marrow and can be mobilized to peripheral blood by the action of certain growth factors, such as G-CSF or VEGF [76,77]. EPCs have been well utilized in the endothelialization of synthetic vessel grafts as well as in vessel engineering [9]. Adipose tissue is another source of stem cells from which ECs and SMCs can be derived. Human adipose progenitor cells have been shown to differentiate towards an endothelial lineage in the presence of VEGF [78]. Adipose stem cells can be harvested, multiplied and handled easily, efficiently and non-invasively, and they have a proliferative capacity comparable to bone marrow mesenchymal cells. Moreover, donor morbidity is considerably reduced, and harvesting requires only local anesthesia and results in a short donor site healing time [79]. Bertanha et al. showed that endothelial cells that differentiate from adipose tissue-derived MSCs by platelet-derived growth factor are able to adhere to decellularized vascular grafts [80]. Krawiec et al. tested the in vivo function of a tissue-engineered vascular graft based on adipose tissue-derived stem cells from high cardiovascular risk populations and illustrated the importance of testing cells from realistic patient populations, such as those with diabetes [81]. The same group also showed that the stromal vascular fraction (SVF) of adipose tissue was an abundant, culture-free cell source for tissue-engineered vascular grafts. The SVF represents a heterogeneous cell population surrounding adipocytes in fat tissue, including mature microvascular endothelial cells [82]. The SVF cells were also capable of being seeded within biodegradable, elastomeric, porous scaffolds, which maintain patency for as long as eight weeks when implanted in the rat aorta [83]. Sundaram et al. constructed a functional vascular graft with iPS cell-derived smooth muscle cells and a synthetic polymer in vitro. After eight weeks in a pulsatile bioreactor system, histological analyses confirmed the presence of layers of calponin-positive smooth muscle cells in a collagen-rich matrix [84]. In addition to decellularized vascular grafts, recellularization has also been investigated in other areas of tissue and whole-organ engineering such as in the cornea, heart, kidney, lung, liver, uterus, ovary and testis [85,86,87,88,89,90,91,92,93,94,95,96,97,98,99,100,101].

### 1.5. Animal Models

When animal models are selected for studies of decellularized vascular grafts, it is important to follow the minimally acceptable criteria relevant to vascular grafts, such as graft diameter, vessel length, vessel accessibility, flow type (high or low), anastomosis style (end-to-end or end-to-side) and suitability to be sutured. Some physiological factors that are different among animal species should be considered, such as thrombogenicity, hemodynamics, hematology and immunogenicity. Other equally important factors relevant to selected animal species are the selection of anesthesia technique, ability to be sterilized, cost, ease of handling, reproducibility of the procedure, long-term remodeling and function [71,102].

Animals that are commonly used in decellularized vascular graft experiments are similar to those used in studies of other types of vascular grafts and include mice, rats, rabbits, dogs, sheep/goats, and pigs. Baboons are rarely used because of ethical concerns [71,102]. For mice or rats, the abdominal aorta or carotid artery interposition models are commonly selected, as these areas are good recipient sites. For rabbits, dogs, sheep, and pigs, the carotid artery, jugular vein or abdominal aorta is commonly selected as a recipient site [71,103]. End-to-end anastomosis is the most frequently used surgical technique for the interposition of grafts if no size discrepancy exists. The strategies by which the results are recorded include a single measurement at the end of the experiment and serial measurements at different time points. Gross examinations can provide information about patency (narrowing or occlusion), dilatation, and thrombosis. Histological examinations can reveal endothelialization, intimal hyperplasia, cellular infiltration in the medial layer, inflammation, calcification and remodeling. Imaging modalities such as Doppler or CT-angiogram can detect patency and flow velocity [102]. 

Additionally, similarity to human physiology is one major factor that is considered when criteria specifically related to translational studies are assessed. Small animals like mice and rats are poor models for long-term evaluation because they lack similarity in vessel size and hemodynamics [71]. However, these animals have advantages such as low cost and ease of maintenance, and they serve as a very useful resource for short-term follow-up (patency, rupture, hemorrhage) and for studies of the mechanisms related to endothelialization and inflammation. Usually, hemodynamic aspects of cardiovascular grafts should be examined in adequate large animal models, such as sheep/goats, pigs, or baboons. However, no in vivo models, which are ideal for testing all criteria, have been reported [102]. Hence, animal models are needed to test specific developmental criteria [104]. Table 1 lists the considerations for the selection of animal models for the testing of decellularized vascular grafts.

## 2. In Vivo Performance of Decellularized Vascular Grafts

### 2.1. Materials and Methods

We searched articles related to the in vivo performance of decellularized vascular grafts in PubMed using the following keywords: animals, blood vessel prosthesis, acellular from 2001 to 2017. We also searched Google Scholar using the following keywords: animals, in vivo, blood vessel prosthesis, decellularization, and acellular from 2001 to 2017. In total, 1742 articles were found (PubMed 92/Google Scholar 1650). Studies on vascular conduits with valves, those used for patch repair, grafts that feature anastomosis to veins, those with no vascular anastomosis and ex vivo models were excluded. Ultimately, 63 articles were included for review.

### 2.2. Cell-Free Decellularized Grafts without Further Treatment

We included 18 articles (Table 2) in which decellularized vascular grafts were studied in animal models (17) and those that assessed these grafts in human trials (1). The source of the vascular graft could be allogenic or xenogenic. The transplantation model included artery to artery, vein to artery or non-artery (ureter or amnion membrane) to artery. Decellularized allogenic vascular grafts with artery to artery transplantation had a patency rate of 83% (at four weeks) to 100% (at 14 months) [59,107,108,109,110,136,137,138]. No dilatation or aneurysm formation was observed in most studies. Histology showed endothelialization occurred at 2–8 weeks post-implantation. Gradual cellular infiltration and SMC repopulation with low levels of calcification were observed. Progressive vascular wall remodeling was noted with longer follow-up (~6 months). However, some degree of intimal hyperplasia was noted [107]. Decellularized allogenic vascular grafts with vein to artery transplantation could also achieve a 100% patency rate [126,127]. But incomplete endothelialization was noted. This finding might be related to the different compositions and structures of arteries and veins, which could lead to biomechanical mismatch in vivo [127]. For decellularized xenogenic artery to artery transplantation, the patency rate was about 45.5% to 100% [7,10,11,105,139]. Nonetheless, histology showed mixed results that could be attributed to different xenogenic transplantation models. With recent advances in decellularization techniques, Negishi et al. showed good endothelialization and cellular infiltration in an HHP-decellularized xenogenic graft model. The luminal surface of the graft showed endothelial cell coverage and cellular infiltration of the medial layer [10]. In vivo performance of decellularized xenogenic non-arterial tissues, such as bovine ureter or human amniotic membrane, were also evaluated in animal models [12,122]. But in a human hemodialysis access trial, Chemla et al. found that a decellularized bovine ureter (SG100) did not have an advantage in graft patency or stability over expanded polytetrafluoroethylene (ePTFE) [5].

### 2.3. Decellularized Vascular Grafts with Biomolecules or Growth Factors

We included 16 articles (Table 3) on the use of biomolecule/growth factor-modified decellularized vascular grafts tested in animal studies. Four studies in which decellularized allogenic grafts were used, 11 studies in which decellularized xenogenic grafts were used and one study with an unknown graft type were included. At present, the patency rates of biomolecule/growth factor-modified decellularized vascular grafts are between 90 and 100%. Modified decellularized vascular grafts that are either allogenic or xenogenic achieved early confluent endothelialization, cell infiltration, vessel wall maturation and even restoration of contractile function [143].

Thebiomolecules/growth factors that were applied for-modified decellularized allogenic/xenogenic vascular grafts included heparin, VEGF, brain-derived neurotrophic factor (BDNF), granulocyte-colony stimulating factor (G-CSF), CCN1 and functional peptide [8,44,46,111,113,123,128,129,143]. Heparin and VEGF improved endothelialization, reduced intimal hyperplasia and enhanced vessel wall remodeling of decellularized vascular grafts in vivo [8,13,114,115,124,128]. G-CSF induced mobilization of circulating EPCs, regenerated the endothelium and inhibited neointimal hyperplasia of the decellularized vascular graft in a rat model [111]. In contrast, Heidenhain et al. found a massive stimulation of giant cells and eosinophils, whereas pseudointimal hyperplasia was significantly increased in genipin-crosslinked, FGF (fibroblast growth factor)/VEGF-coated decellularized rat artery in vivo [112]. Recently, Lee et al. found that anti-hypertensive drug, losartan, could preserve the medial layer and decrease elastin degradation of decellularized allogenic vascular grafts in a rat model [48]. CCN1 could enhance endothelialization, vessel wall remodeling and neovascularization of decellularized xenogenic vascular graft in vivo [46]. Mahara et al. found that coating with a hetero-bifunctional peptide enhanced endothelialization and restoration of function when decellularized xenogenic vascular grafts were used [143].

### 2.4. Decellularized Vascular Grafts with Recellularization

We included 20 articles (Table 4) on the use of recellularized vascular grafts in animal studies. Decellularized matrices in which the luminal surface does not contain an endothelial cell (EC) lining carry a substantial risk for thrombosis when exposed directly to blood flow [7]. Therefore, in recent years, much work has focused on the generation of tissue-engineered vascular grafts by recellularizing scaffolds with host vascular cells prior to implantation [9,131]. The decellularized vascular grafts that are included were either allogenic or xenogenic. The cell source includes autologous, allogenic and xenogenic cells. The cell type used for seeding includes ECs or EPCs only, ECs/SMCs or genetically modified ECs or stem cells. For decellularized allogenic vascular grafts with recellularization using autologous/allogenic ECs or EPCs, the results showed better patency rate than control, confluent endothelium formation and SMCs repopulation [27,116,140,144], But Zhou et al. found that decellularized canine arterial matrix recellularized with autologous EPCs had a small amount of neointimal hyperplasia at the mid-portion and anastomotic sites of the graft. [27,116,132] Decellularized allogenic vascular grafts with dual autologous cell (ECs/SMCs) recellularization showed good patency rate, complete endothelialization, decreased intimal hyperplasia, cellular repopulation and tissue maturation of vascular grafts in vivo [31,131,133,134,141,145]. G-CSF has been further demonstrated to enhance the extensive endothelium regeneration of dual cell recellularizing vascular grafts in canine abdominal aorta transplantation model [43,131]. With respect to decellularized allogenic vascular grafts with xenogenic cell recellularization, Wong et al. showed the presence of seeded ECs/SMCs (derived from human iPS cells) along with host endothelial cells and smooth muscle cells on decellularized mouse vascular grafts in vivo [106].

With respect to decellularized xenogenic vascular grafts with recellularization, autologous EPC-seeded decellularized porcine vascular grafts exhibited confluent endothelialization, neo-intimal and neo-medial reconstitution and gained functional SMCs in vivo [9,56]. But Ma et al. observed dilatation of decellularized fetal pig vascular grafts with allogenic EC recellularization in a canine carotid artery model [56]. Decellularized vascular grafts recellularized with genetically modified cells, like A20 gene, showed anti-atherosclerosis in vivo [117]. Vascular grafts recellularized with TSK2 gene knock down endothelial cell exhibited better endothelialization and mural cell recruitment [118]. Mcllhenny et al. created a novel tissue-engineered blood vessel by seeding a decellularized human saphenous vein allograft with autologous ASCs differentiated into endothelial-like cells. Successful implantation and patency up to eight weeks in a rabbit aorta model were observed after transfection of the endothelial nitric oxide synthase (eNOS) gene to produce NO, though intimal hyperplasia was also noted [125]. Interestingly, some studies traced the allogenic donor cells in cell-seeded decellularized vascular grafts after implantation and found that the number of donor cells decreased gradually as they were replaced by host cells. The role of allogenic donor cells might be to exert paracrine effects that in turn recruit host cells [69,131]. At present, in animal studies, recellularized vascular grafts can achieve a 60–100% patency rate. In a proof of concept study in humans, Olausson et al. used recellularized vascular grafts in two patients. However, one gained 100% patency up to 21 months, but the other patient required a secondary graft seven months after surgery due to thrombosis [148]. 

### 2.5. Variants of Decellularized Vascular Grafts

We included 9 studies in this category (Table 5). The variants of decellularized vascular grafts were either recellularized or not. Some studies developed cell-based vascular grafts in vitro that were then decellularized before implantation. The results of these studies showed patent lumen with endothelialization in vivo [28,142]. Another technique is developing tissue engineered vessels with biomimetic perfusion system. The engineered vessels were then decellularized, re-seeded with cells and were implanted in vivo. The results showed less neointimal hyperplasia, but increased neo-endothelialization, smooth muscle repopulation, and tissue regeneration of vascular grafts in vivo [20,28,119,142,147]. Another novel technique that has been applied to decellularized vascular grafts results in “hybrid” vascular grafts. The goal of this kind of vascular graft is to solve the shortcomings of synthetic polymers such as thrombosis, intimal hyperplasia, calcification, chronic inflammation, no growth potential, and compensatory alterations in the mechanical properties of allogeneic vascular grafts during the decellularization process. The in vivo results of hybrid vascular grafts also showed a high patency rate, increased host cell infiltration, decreased inflammation, and successful prevention of vasodilation and aneurysm formation, while the inflammatory cell infiltration was reduced [69,120,121,146].

## 3. Discussion

Cell-free decellularized grafts without further treatment are the basis for developing other kinds of decellularized vascular grafts. They have advantages of saving time for biomolecule/growth factor coating, cell culture and material process. The disadvantages of cell-free decellularized grafts are the risk of incomplete endothelialization and early intimal hyperplasia in vivo. There are many choices of biomolecules or growth factors that could be applied in decellularized vascular grafts. The advantages of decellularized vascular grafts with biomolecules or growth factors include increased endothelialization, cellular infiltration and vascular wall remodeling in vivo. The disadvantages of decellularized vascular grafts with biomolecules or growth factors are the time-consuming nature of coating and additional therapy, like G-CSF injection or oral medication (losartan), needed. Recellularization technique has the advantage of providing a cellular interface between grafts and host blood flow. It could also improve endothelialization, reducing inflammatory reaction, increased cellular infiltration, and vascular wall remodeling of decellularized vascular grafts. The major drawback in recellularized vascular grafts is time consuming in graft preparation and high cost. Hybrid-decellularized grafts have advantages of improving biochemical or mechanical properties in cell-free decellularized grafts and thus in vivo performance. However, there is the disadvantage of high cost. 

## 4. Conclusions

In this review, we summarized the current strategies for the production of decellularized vascular grafts in pre-clinical and clinical studies. The decellularized vascular grafts included those without further treatment, those with bound biomolecules/growth factors, those that underwent recellularization (EPCs, ECs, SMCs, stem cells or genetically modified cells), and other variants (decellularization of cell-based TEVGs, hybrid TEVGs). The current results are encouraging and showed both short-term and long-term promise for the use of decellularized vascular grafts as vascular substitutes in animals. The implanted grafts became functional, as evidenced by endothelialization, biocompatibility, remodeling, and matched mechanical properties over time. Endothelial cells resurface the luminal site, and smooth muscle cells repopulate the medial layer with new collagen synthesis. However, it should also be noted that the first allogeneic tissue-engineered vascular graft implanted in humans for hemodialysis access resulted in high thrombotic failure rates. Moreover, unfavorable results in graft patency and stability were obtained in a recent clinical trial for decellularized vascular grafts [5,148,149]. The current challenges to developing engineered functional and implantable decellularized vascular grafts include immune rejection of allogenic cells, autologous cell senescence, long culture time in bioreactors, and the high cost of recellularization methods. More studies are required to cross the current gap from the lab bench to the clinical application of decellularization-based vascular grafts.

## Figures and Tables

**Table 1:** Consideration in selecting animal models for decellularized vascular grafts.

Animal	Recipient Site	Diameter	Anastomosis	Pro	Con	Reference
Mouse	Abdominal aorta	1 mm	End-to-end	Easily handle; Easily anesthesia; Easily maintain; Similar hemostatic mechanism to human; Less maintenance costs	Small size; Need microsurgery; High mortality rate; Learning curve	[105,106]
Rat	Carotid artery	1–3 mm	End-to-end	Similar to mouse	Similar to mouse	[7,10,27,44,48,107,108,109,110,111,112,113,114,115,116,117,118,119,120,121]
	Abdominal aorta	1–3 mm	End-to-end or End-to-side			
Rabbit	Carotid artery	1–4 mm	End-to-end	Easily handle; Easily anesthesia; Similar hemostatic mechanism to human; Bilateral transplantation; Less maintenance costs	Relative small size	[11,122,123,124,125]
	Abdominal aorta	1–4 mm	End-to-end			
Canine	Carotid artery	3–5 mm	End-to-end or End-to-side	Easily handle; Easily access vessel due to thin skin; Selection of vascular size; Similar cardiovascular physiology to human; Tolerance to anesthesia	Relatively hypercoagulability; Higher maintenance costs; Availability restriction	[8,12,43,56,126,127,128,129,130,131,132,133,134,135]
	Abdominal aorta	3–5 mm	End-to-end			
Sheep/Ovine/Goat	Carotid artery	4–6 mm	End-to-end	Easily handle; Long neck; Similar cardiovascular physiology to human; Coagulation system similar to human; Low costs	Higher incidence of clotting	[9,13,46,59,69,136,137,138,139,140,141,142]
	Pulmonary artery	4–6 mm	End-to-end			
	Aorta/descending aorta	4–6 mm	End-to-end			
	Femoral artery	4–6 mm	End-to-end			
Porcine	Carotid artery	4–6 mm	End-to-end or End-to-side	Low purchase costs; Similar cardiovascular morphology & physiology to human	Difficulties in handling; Less tolerance of anesthesia; Higher maintenance costs; Rapid growth	[20,31,143,144,145,146]
	Femoral artery	4–6 mm	End-to-end			
Baboon	Axilla-Brachial artery	4–6 mm	End-to-end	Similar cardiovascular morphology, physiology and thrombotic mechanism to human; Greater availability of cellular markers, non-invasive imaging technologies, thrombosis evaluation assays; Limited fibrous encapsulation	Difficulties in handling; Higher maintenance costs; Ethical concerns	[28,147]

**Table 2:** Summary of in vivo performance of cell-free decellularized grafts without further treatment since 2001.

Author	Tissue Source	Animal Model	Patency Rate (%)	Histology (Endothelialization/Remodeling)
Decellularized allogenic vascular graft				
Bergmeister (2005) [59]	Ovine carotid artery	Sheep carotid artery	?	Recellularization at 6 weeks; A significantly thicker neointima and neovascularization; ECM reconstitution;
Boerboom (2002) [136]	Goat carotid artery	Goat carotid artery	100% at 3 and 6 months	ECs+ at intima, SMCs+ at media, Fibroblast at adventitia; Less intima thickness at midportion
Hilbert (2004) [137]	Goat carotid artery	Goat carotid artery	100% at 7 months	Neointima formation, ECs+; Host cell migration; No blood vessel ingrowth
Ketchedjian (2005) [138]	Sheep pulmonary aorta	Sheep descending aorta or pulmonary artery	? (compared to cryopreservation group)	Migration of smooth muscle cell through matrix; without calcification
Hwang (2011) [107]	Rat abdominal aorta	Rat abdominal aorta	100% at 8 weeks	More endothelialization at 8 weeks but less than normal tissue; Intimal hyperplasia progress from 2–8 weeks; Cellularity increased gradually
Assmann (2013) [108]	Rat thoracic aorta	Rat abdominal aorta (end-to-side; iv. heparin 300 IU/kg)	100% at 8 weeks (postoperative recovery rate 80%)	Local intimal hyperplasia and accumulation of SMCs; Microcalcification was observed
Sakakibara (2014) [109]	Rat artery	Rat abdominal aorta	Patent up to 14 months	Complete ECs cover at 5 weeks; Formation of tunica intima and tunica media (SMCs); Contractile function observed at 12 months
Nagaoka (2014) [110]	Rat abdominal aorta	Rat abdominal aorta	83% at 4 weeks (silastic tube 0%)	Complete endothelialization at 4 weeks
Schaner (2004) [126]	Canine jugular vein	Canine carotid artery	100% at 2 weeks	No significant graft dilation, rupture or anastomotic false aneurysm
Martin (2005) [127]	Canine jugular vein	Canine carotid artery	100% at 8 weeks	Fibrin layer was noted; Endothelization only at perianastomosis sites; Smooth muscle cell repopulated at 8 weeks
Decellularized xenogenic vascular graft				
Clarke (2001) [12]	Bovine ureter	Canine aorta	100% at 10 months	ECs at luminal site and SMCs at media
Costa (2004) [139]	Porcine artery	Sheep right ventricle outflow tract	100% at 3 and 5 months	Less calcium content than homografts; Progressively repopulated by autologous cells
Lopez-Soler (2007) [105]	Ovine arterial tissue	SCID Mouse abdominal aorta	100% at 35 days (control: silastic tube)	Non-occlusive wall thrombus and neointimal hyperplasia; Evidence of cellular in-growth
Gui (2009) [7]	Human umbilical artery	Nude rat abdominal aorta (iv heparin. 1000 IU/kg)	45.5% at 8 weeks	Thrombosis at proximal site; slight intimal hyperplasia at 8 weeks; SMCs repopulated; Neo-collagen formation at media
* Chemla (2009) [5]	Bovine ureter (SG100)	Human hemodialysis access		SG 100 were not seen better in either patency or stability; No significant differences in primary patency, assisted primary patency and secondary patency;
Xiong (2013) [11]	Porcine saphenous artery	Rabbit carotid artery	60% at 1 month and 50% at 3 months.	No intimal hyperplasia; Endothelialization increased from 1–3 months; SMCs at media;
Negishi (2015) [10]	Porcine radial artery	Rat artery	100% at 2 weeks	Luminal surface was covered by recipient endothelial cells; Arterial medium was fully infiltrated with recipient cells
Amensag (2017) [122]	Human amnion membrane	Rabbit carotid artery	100% at 4 weeks	No significant decrease in diameter and blood flow; Midpoint wall thickness increased significantly

*: Human clinical trial.

**Table 3:** Summary of in vivo performance of biomolecule/growth factor-modified decellularized vascular grafts since 2001.

Modification	Animal Model	Patency Rate (%)	Histology (Endothelialization/Remodeling)	Reference
Heparin	Xenogenic transplantation	92~100% at 2–6 months	Early confluent ECs lining; Reduce intimal hyperplasia; SMCs repopulated; Less inflammation	[8,114,115,123,124,129,130]
VEGF/Heparin/FGF	Allogenic/xenogenic transplantation	~95% at 6 months	Complete endothelialization; Slight intimal hyperplasia; SMC infiltration; Restore vessel wall structure and contractile function	[13,112,128]
G-CSF/Heparin	Allogenic transplantation	~95% at 6 months	Early endothelialization; Smaller hyperplastic neointima at mid-portion and distal portion; Superior cellular and ultrastructural preservation	[111,113]
BDNF/Collagen	Allogenic transplantation	90% at 8 weeks	Confluent endothelialization; Elastic fibers maintain integrity; CD31+, SMA+ cells was noted;	[44]
CCN1	Xenogenic transplantation	100% at 14 weeks	Complete endothelialization; Multiple layers of SMCs; Supported neovascularization; Enhancing immunologic tolerance	[46]
Hetero-bifunctional peptide (collagen-binding region and the integrin α4β1 ligand)	Xenogenic transplantation	83.3% at 3 weeks	Clear luminal surface without thrombosis; Pulsatile function at 3 weeks	[143]
Losartan	Allogenic transplantation	100% at 8 weeks	A significantly lower medial elastic fragmentation; Superior medial layer preservation; Relatively more normal appearing intimal cellular morphology	[48]

**Table 4:** Summary of in vivo performance of recellularized-decellularized vascular grafts since 2001.

Author	Tissue Source	Cell Type	Bioreactor	Cell Number or Density	Animal Model	Patency Rate (%)	Histology (Endothelialization/Remodeling)
Decellularized allogenic vascular graft with autologous EC or EPC recellularization							
Teebken (2001) [144]	Porcine carotid artery	Autologous EC	?	?	Porcine carotid artery	54% and 71% at 1 and 4 months	Superior patency to polydioxanone prostheses but inferior to the arterial autograft.
Leyh (2006) [140]	Ovine pulmonary artery	Autologous EC	12 h, 0.1 rpm; total 3 cycles	1 × 10^6^	Ovine pulmonary artery (iv. heparin 400 IU/kg)	100% at 6 months (seeded and non-seeded)	Cell infiltration and extracellular matrix similar to native artery in cell seeding group; Degeneration change was noted at control group
* Zhou (2012) [132]	Canine carotid artery	Autologous EPC	Yes	?	Canine carotid artery	95% at 3 months (control: 60%)	Smaller hyperplastic neointima area at mid-portion and anastomotic sites
Decellularized allogenic vascular graft with allogenic EC recellularization							
Borschel (2005) [27]	Rat Iliac artery	Allogenic ECs	No	10^6^/100 μL	Rat femoral artery	89% at 4 weeks (control 29%)	Complete ECs at lumen; SMCs repopulated vessel wall; Mechanical properties comparable to native vessel
Dall’Olmo (2014) [116]	Rat iliac artery	Allogenic ECs	No	4 × 10^5^/cm^2^	Rat abdominal aorta	100% at 3 months	ECs completely covered luminal surface at 1 month; Vascular wall remodeling at 3 months
Decellularized allogenic vascular graft with dual cell recellularization							
Cho (2005) [131]	Canine carotid artery	Autologous bone marrow –derived cells differentiated EC, SMC	No	EC (1 × 10^7^ cells/mL) SMC (3 × 10^7^ cells/mL	Canine carotid artery	100% at 8 weeks (non-seeded occluded at 2 weeks)	Endothelium regeneration; Regeneration of three layer structure; Bone marrow derived cells were detected
# Cho (2006) [43]	Canine abdominal aorta	Autologous bone marrow-derived cells different to EC, SMC	?	?	Canine abdominal aorta	100% (both G-CSF and non G-CSF groups) at 8 weeks	Endothelium formation was more extensive in the G-CSF-treated graft; Intimal hyperplasia was significantly reduced in the G-CSF-treated grafts
Narita (2008) [133]	Canine ureter	Canine EC & myofibroblast	?	?	Canine carotid artery	100% at 6 months (control: 0%)	Non-seeded & PTFE grafts become occluded within a week
Yang (2009) [134]	Canine carotid artery	Autologous ECs and SMCs	Yes	?	Canine carotid artery	100% at 6 months	Similar macroscopic appearance to that of native vessels; High cell density and development of a highly organized structure of ECM.
Zhao (2010) [141]	Ovine carotid artery	Autologous MSC derived EC and SMC	No	SMC (2 × 10^7^/mL); EC (1 × 10^7^/mL)	Sheep carotid artery	100% at 2 & 5 months	The existence of endothelium, smooth muscle and the presence of collagen and elastin both at 2 and 5 months; The MSC derived cells survived and contributed to the vascular tissue regeneration
Dahan (2017) [31]	Porcine carotid artery	Allogenic EC and SMC	50 mL/min and 80 mmHg	0.5–1 × 10^5^ cells/cm^2^	Porcine carotid artery (iv. heparin 100 IU/kg)	100% at 6 weeks (control: 100% but ~20% lumen stenosis)	No intimal hyperplasia, no thrombosis; Lack of neutrophils, macrophages, multinucleated giant cells, and B cells within these grafts; Well-organized host-recruited SMCs were observed in the media layer with a continuous monolayer of ECs on the lumen
Sagban (2011) [135]	Canine vein	EPCs from bone marrow and SMCs	?	?	Canine carotid artery	90% at 3 months	A complete incorporation in the surrounding tissue; No aneurysm degeneration
Neff (2011) [145]	Porcine carotid artery	Autologous EC/SMC	9.9 dyne/cm^2^~13.2 dyne/cm^2^	EC (2 × 10^6^/mL); SMC (5 × 10^5^/mL)	Porcine femoral or carotid artery (iv. heparin 100 IU/kg)	100% at 4 months (both dual seeding and EC only)	More tissue maturation in dual-seeding group; More SMCs infiltration in dual-seeding group; Better contractile function in dual-seeding group
Wong (2015) [106]	Mouse aorta	Human iPS cell differentiated ECs & SMCs	20–35 mL/min for 24 h	5 × 10^5^	Mouse carotid artery	60% at 3 weeks	Presence of human partially induced stem cell-derived; Endothelial/smooth muscle cells and host macrophage infiltration at 3 weeks
Decellularized xenogenic vascular graft with recellularization							
Kaushal (2001) [9]	Porcine iliac artery	Autologous EPC	1–25 dyne/cm^2^	1 × 10^5^ cell/cm^2^	Sheep carotid artery	100% at 130 days (control: 25% at 15 days)	Confluent endothelialization; EPCs covered approximately 80% and 10% of the graft lumen, at 15 and 130 days; Functional SMCs at 130 days
Ma (2017) [56]	Fetal pig aorta	Allogenic ECs	20–60 mL/min	3 × 10^6^/mL	Canine carotid artery	100% at 6 months	No stenosis, expansion or thrombosis; Neointima (ECs) and neomedia (SMCs) reconstitution
Gene-modified cell recellularized vascular graft							
Zhu (2008) [117]	Porcine carotid artery	Allogenic A20- regulated EPC differentiated EC and SMC	SMC (pulsatile radial stress 150 beats/min) EC (1–3 × 10^−2^ N/m^2^)	SMC (10^7^/mL); EC (3 × 10^6^/mL);	Rat carotid artery	100% at 6 months (control: 0%)	No inflammatory response; No stenosis or intimal thickening; Fewer apoptotic cells.
Mclhenny (2015) [125]	Human saphenous vein	Autologous ASC (transfected Ad-eNOS gene)	1.5 dynes/cm^2^ at 0.2 Pa/day	2 × 10^5^ cells/cm^2^	Rabbit abdominal aorta (iv. heparin 100 IU/kg)	100% at 2 months (control: 100% but fibrin+)	An intact luminal cell layer without evidence of fibrin formation; Wall thickening was noted
Kristofik (2017) [118]	Rat thoracic aorta	Allogenic TSP2 KO cells	Yes	50 µL of cells (at a concentration of 3 × 10^6^ cells/mL) twice	Rat abdominal aorta	100% at 4 weeks (better than control)	Improved endothelial and mural cell recruitment; Decreased failure rate compared to control grafts
Human clinical trial							
Olausson (2014) [148]	Human iliac or mammary vein	Autologous ECs and SMCs	Heparin: 50 IU/mL PBS for 2 h	Whole blood: 2 mL/min for 48 h; followed by 4 days EC and 4 days SMC medium perfusion	2 Pediatric patients (portal veins)	100% at 21 months in first patients; The second patient received another graft at 7 months (under anticoagulation)	A proof of concept in using tissue engineered vascular graft for clinical patients

*: Heparin coated vascular graft; #: with G-CSF injection.

**Table 5:** Summary of in vivo performance of variant-decellularized vascular grafts since 2001.

Author	Tissue Source	Recellularization	Cell Type	Bioreactor	Cell Number or Density	Animal Model	Patency Rate (%)	Histology (Endothelialization/Remodeling)
Decellularized Cell-based TEVGs								
Quint (2011) [20]	TEVG from allogenic SMC	Yes	Autologous EPC or EC	15 dynes/cm^2^ for 24 h.	2 × 10^5^/cm^2^	Porcine carotid artery (end to side; iv. heparin 100 IU/kg)	100% at 30 days; (control 16.7%)	Less intimal hyperplasia and fewer cell infiltration
Dahl (2011) [28]	Polyglycolic acid with human or canine SMCs	Yes	Canine EC (for small diameter graft)	?	?	Baboon AV shunt (large diameter); Canine peripheral artery (small diameter)	88% up to 3 months in baboon; 83% up to 1 month in dog	No dilatation, calcification, and intimal hyperplasia; Endothelialization at both near anastomotic sites and midgraft in both grafts; SMC infiltration by 6 months in baboon model and by 1 month in canine model; No significant collagen degradation; Mechanical robust
Syedain (2014) [142]	TEVG from Ovine fibroblast/Fibrin gel	No	No	No	No	Ovine femoral artery	?	Endothelialization was complete by 24 weeks with elastin deposition evident; No evidence of dilatation or mineralization; Mid-graft lumen diameter was unchanged; Extensive recellularization occurred, with most cells expressing α-SMA.
Tondreau (2016) [119]	TEVG from human fibroblast	No	No	No	No	Rat abdominal aorta	83.3% at 6 months	Neointima (ECs) and neomedia (SMCs) at 6 months
Syedain (2017) [147]	TEVG from human fibroblast/fibrin gel	No	No	No	No	Baboon axillary-cephalic; axillary-brachial (antiplatelet)	83% and 60% at 3 and 6 months	No calcifications, loss of burst strength, or outflow stenosis; Developing endothelium at lumen and SMCs
Hybrid decellularized vascular graft								
Hinds (2006) [146]	SIS/Fibrin/Elastin	No	No	No	No	Porcine carotid artery	?	Significantly longer average patency times than ePTFE; Low thrombogenicity; Cellular repopulation
Row S (2016) [69]	SIS/fibrin gel	Yes	Allogenic EC+SMC	5 rpm	(2 × 10^6^ cells/mL, 5 mL/graft)	Ovine carotid artery (iv heparin 100 IU/kg)	100% at 3 months (control EC only: 100%)	Cell infiltration at whole layer; Extracellular matrix mature and remodel into functional SMCs; The donor cells were gradually replaced by donor cells; Contractile function+
* Gong (2016) [120]	Rat aorta/Polycaprolactone	No	No	No	No	Rat abdominal aorta (end-to-side; iv. heparin 300 IU/kg)	100% at 6 weeks	No evidence of stenosis and thrombosis; Fiber structure intact; Endothelialization+, SMA+; Reduce extravascular inflammatory cell infiltration
Negishi (2017) [121]	Porcine aorta/fibrin gel	No	No	No	No	Rat carotid artery	100% at 3 weeks	Cell attachment at luminal surface and cell infiltration at luminal wall

*: Heparin coated.
